# Bilateral coronary ostial disease following mediastinal irradiation: a case report

**DOI:** 10.4076/1757-1626-2-7792

**Published:** 2009-08-25

**Authors:** Salman Waqar, Rajwinder Jutley, Richard Mount, Pradip Sarkar

**Affiliations:** Department of Cardiothoracic Surgery, Northern General HospitalSheffield Teaching Hospitals NHS Trust, Sheffield, S5 7AUUK

## Abstract

**Introduction:**

Ostial coronary artery disease is rare with a reported incidence of 0.07 to 0.25% in all patients undergoing angiography. It has a strong association with previous mediastinal irradiation, which induces specific histological changes distinct from atherosclerotic lesions. The radiation also affects the myocardium and surrounding structures, which can alter the surgical approach.

**Case presentation:**

We present a case of a 62-year-old female who developed bilateral ostial coronary artery stenosis 32 years following therapeutic radiotherapy for Hodgkin’s disease. She underwent successful coronary artery bypass surgery using a combination of arterial and venous conduits. Postoperatively she developed a clinical picture of diastolic impairment not detected pre-operatively. She was managed appropriately and made a successful recovery.

**Conclusions:**

This case highlights the cardiac pathology associated with mediastinal irradiation, which should be suspected during surgical assessment, especially in long-term survivors. It heightens the surgeon’s awareness so a more thorough evaluation of coronary anatomy, ventricular function and potential conduits is made prior to surgery.

## Introduction

Ostial coronary artery disease is rare with a reported incidence of 0.07 to 0.25% in all patients undergoing angiography [1]. It has a strong association with previous mediastinal irradiation, which induces specific histological changes distinct from atherosclerotic lesions. The radiation also affects the myocardium and surrounding structures, which can alter the surgical approach.

## Case presentation

**Figure 1.**
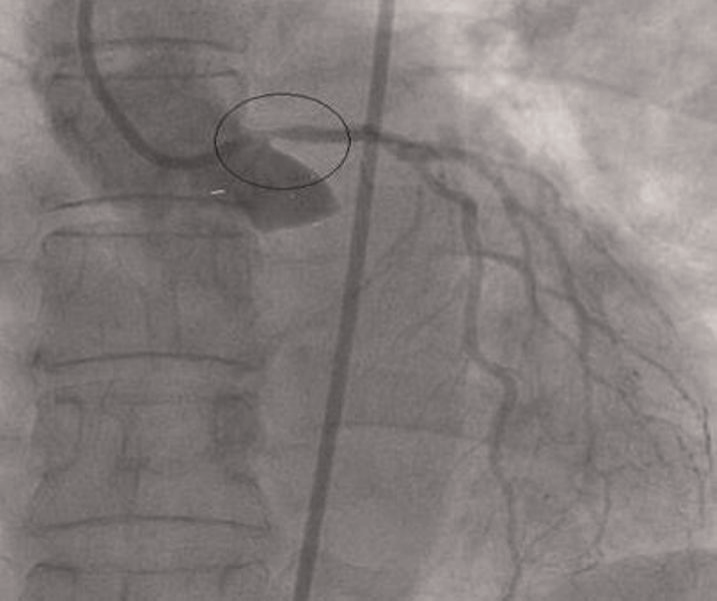
Injection of the left coronary system showed ostial coronary disease associated with a pressure drop across the lesion.

**Figure 2.**
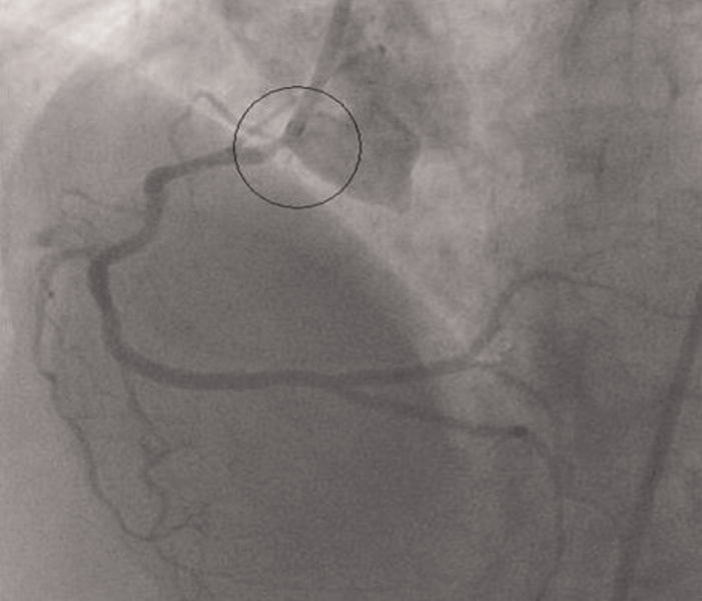
The right coronary system also had similar features on injection of the contrast.

A 62-year-old British white Caucasian female presented with a 2-year history of Canadian Cardiovascular Society (CCS) Class II exertional angina and mild dyspnoea. Her medical history consisted of hypertension, hypercholestrolaemia and Hodgkin’s disease 32 years previously for which she underwent radiotherapy to the chest and pelvis. The total radiotherapy dose delivered was 5330 rads (cGy). She underwent coronary angiography following a strongly positive exercise test. This showed severe (>90%) stenosis at the origin of left main stem associated with a significant pressure drop across the lesion. There was also significant stenosis in the proximal circumflex artery, intermediate artery and proximal left anterior descending (LAD) artery (Figure 1). Injection of the right coronary system demonstrated severe ostial stenosis (>90%) again associated with a pressure drop across the lesion (Figure 2). Her left ventricular function was preserved on ventriculogram. The patient underwent urgent CABG using saphenous vein to bypass the right coronary artery (RCA), a radial artery graft to the obtuse marginal (OM) and left internal mammary artery (LIMA) to the mid-LAD. She was weaned from cardiopulmonary bypass (CPB) without inotropic support. Her initial recovery was unremarkable, and she had a fast-tracked extubation. Within a few hours, however, she developed signs of low cardiac output syndrome with oliguria. Fluid challenges were administered resulting in rapidly raised central venous pressures (CVP) and dyspnoea secondary to pulmonary oedema. Invasive monitoring with a pulmonary artery wedge catheter (PAWC) demonstrated high filling pressures (pulmonary artery wedge pressure of 20 mmHg) with a low cardiac index of 1.8. The features were suggestive of severe diastolic dysfunction. Inotropic support with adrenaline was commenced with good clinical response. She improved with circulatory support and continuous positive airway pressure (CPAP) over the following 3 days and was discharged home 8 days later.

## Discussion

Radiotherapy is an established treatment modality for a variety of tumours including Hodgkin’s disease. Radiotherapy is also used as adjuvant treatment in breast cancer. Both breast cancer and Hodgkin’s disease account for the most important causes of radiation-induced cardiac damage with the latter resulting in a higher relative risk estimate for fatal cardiovascular events (2.2 to 7.2) [2]. The risk correlates with younger age at irradiation, length of follow-up and dose volume used [3].

Cardiac damage can manifest in a variety of ways especially if >65% of the heart has been irradiated [4]. Clinical presentation ranges from most commonly pericarditis to valvular dysfunction, conduction abnormalities and myocardial infarction [4,5]. A rare complication of mediastinal irradiation is the development of coronary artery disease [6,7]. The pattern of disease is unusual in that it tends to affect the coronary ostia, presumably due to their relatively central location within the radiation field. Histologically, radiation-induced coronary lesions vary from atherosclerotic disease although it has been suggested that both pathologies may act in synergy whereby atherosclerosis is accelerated if risk factors such as hypercholesterolaemia are present [8]. The intimal plaque is similar in both radiation-induced disease and atherosclerosis. Virani *et al* demonstrated that characteristically with radiation damage there is medial thinning and adventitial fibrosis [9]. Other histological features include intimal foam cell collections with calcification and necrosis within the central core of the plaque. Irradiation damage to the heart may extend beyond the coronary arteries. A surgical autopsy study by Vienot and Edwards at the Mayo Clinic on 27 patients with previous mediastinal radiation showed that 71% of cases had radiation injury to the valves with the mitral valve the most affected. 63% of cases had radiation-induced fibrosis most severe in patients who received a radiation dose greater than 3000 rad (cGy) radiation dose [10]. It is likely the diastolic dysfunction observed in our patient was secondary to radiation induced subendocardial fibrosis as she received a-cumulative-dose of 5330 rad (cGy). Although theoretically less frequent with modern techniques of radiotherapy that employ lower doses and cardiac shielding, fibrosis would have prevented myocardial stretch with hindrance of Starling’s forces. This dysfunction was not apparent on reviewing the pre-operative ventriculogram, which tends to evaluate systolic function.

The Internal Mammary Arteries (IMA) may also be included within the radiation field and damaged thereby precluding its use for coronary bypass grafting [11]. However, in a comprehensive review of forty-nine patients with previous mediastinal irradiation who underwent elective coronary bypass surgery using at least one IMA, Nasso *et al* found no incidence of graft failure [12]. Intra operative mammary flow rates assessed by Doppler showed no difference between irradiated and non-radiated patients. The group did, however, suggest the use of skeletonisation during conduit harvesting due to the abnormally high incidence of fibrous tissue around the vessel to achieve maximum length.

Our report highlights the complexities in dealing with patients with previous mediastinal irradiation, more so when historically higher doses with lack of cardiac shielding were employed. In support of the observations made by Fuzellier *et al*, we recommend a multi-disciplinary approach when managing such patients, especially if the patient has previously received a radiation dose of more than 3000 rad (cGy) [7]. It is important that patients are carefully assessed for ostial coronary lesions, preferably to also assess the internal mammary arteries and echocardiography performed routinely to evaluate ventricular function. As long-term survivors following mediastinal irradiation (especially for Hodgkin’s disease) are at higher risk of cardiovascular fatality, particular attention must be paid to this subset of patients. Surgery is the treatment of choice and the use of the IMA over saphenous vein grafts should be encouraged as it provides a prognostic benefit [13].

## Abbreviations

CABG: coronary artery bypass graft
CCS: canadian cardiovascular society
CPAP: continuous positive airway pressure
CPB: cardio-pulmonary bypass
CVP: central venous pressure
LAD: left anterior descending
LIMA: left internal mammary artery
OM: obtuse marginal
RCA: right coronary artery
